# YAP inhibits autophagy and promotes progression of colorectal cancer via upregulating Bcl-2 expression

**DOI:** 10.1038/s41419-021-03722-8

**Published:** 2021-05-07

**Authors:** Lan Jin, Yunhe Chen, Dan Cheng, Zhikai He, Xinyi Shi, Boyu Du, Xueyan Xi, Yujing Gao, Yang Guo

**Affiliations:** 1 Department of Immunology, School of Basic Medical Sciences, Hubei University of Medicine, 442000 Shiyan, Hubei P. R. China; 2 Hubei Key Laboratory of Embryonic Stem Cell Research, Hubei University of Medicine, 442000 Shiyan, Hubei P. R. China; 3 Key Laboratory of Fertility Preservation and Maintenance of Ministry of Education, Department of Biochemistry and Molecular Biology, School of Basic Medical Sciences, Ningxia Medical University, Yinchuan, P. R. China

**Keywords:** Oncogenes, Tumour biomarkers

## Abstract

Colorectal cancer (CRC) is one of the most aggressive and lethal cancers. The role of autophagy in the pathobiology of CRC is intricate, with opposing functions manifested in different cellular contexts. The Yes-associated protein (YAP), a transcriptional coactivator inactivated by the Hippo tumor-suppressor pathway, functions as an oncoprotein in a variety of cancers. In this study, we found that YAP could negatively regulate autophagy in CRC cells, and consequently, promote tumor progression of CRC in vitro and in vivo. Mechanistically, YAP interacts with TEAD forming a complex to upregulate the transcription of the apoptosis-inhibitory protein Bcl-2, which may subsequently facilitate cell survival by suppressing autophagy-related cell death; silencing *Bcl-2* expression could alleviate YAP-induced autophagy inhibition without affecting YAP expression. Collectively, our data provide evidence for YAP/Bcl-2 as a potential therapeutic target for drug exploration against CRC.

## Introduction

Colorectal cancer (CRC) is a common gastrointestinal malignancy, which is the third most commonly diagnosed cancer in males and the second one in females worldwide^1^. Yes-associated protein (YAP), a transcriptional coactivator, is a downstream effector in the Hippo signaling pathway, which plays an important role in controlling organ size, regulating self-renewal, and differentiation of stem cells^2–4^. By inducing cancer stem cell attributes, proliferation, chemoresistance, and metastasis, YAP functions as an oncoprotein and is essential for cancer initiation or progression of most solid tumors, such as cholangiocarcinoma, ovarian cancer, hepatocellular carcinoma, and gastric cancer^5–9^. In colorectal cancer, it has been shown that high expression of YAP is highly associated with increased cell proliferation and poor prognosis^10^.

Autophagy is an evolutionarily conserved cellular degradation system for maintaining intracellular homeostasis^11^. Its function varies significantly in cancer, depending on the cancer type and distinct cellular context. Under appropriate conditions, autophagy protects tumor cells from death and results in the occurrence of chemoresistance by removing or mitigating harmful stimuli. Conversely, excessive or prolonged autophagy plays tumor-suppressive roles by inducing autophagy-related cell death, which is independent of, or in parallel with apoptosis upon certain cellular conditions^12–15^. The role of autophagy in the pathobiology of CRC is intricate and contradictory, and the mechanisms have not been well documented.

Recent work has shown that expression of YAP is elevated in CRC tissues compared with that in normal tissues^16^. Furthermore, YAP appears to be important in inhibiting autophagy in thyroid cancer cell lines^17^. In this study, we aimed to reveal the effect of YAP on autophagy in colorectal cancer and elucidate the possible molecular mechanism. Our findings suggest that YAP promotes the in vivo growth of human CRC cells, and inhibits autophagy both in vitro and in vivo; *Bcl-2* expression could be directly upregulated by YAP at the transcriptional level; correspondingly, silencing *Bcl-2* expression could alleviate YAP-induced autophagy inhibition.

## Results

### YAP inhibits autophagy in human CRC cells

**Fig. 1:**
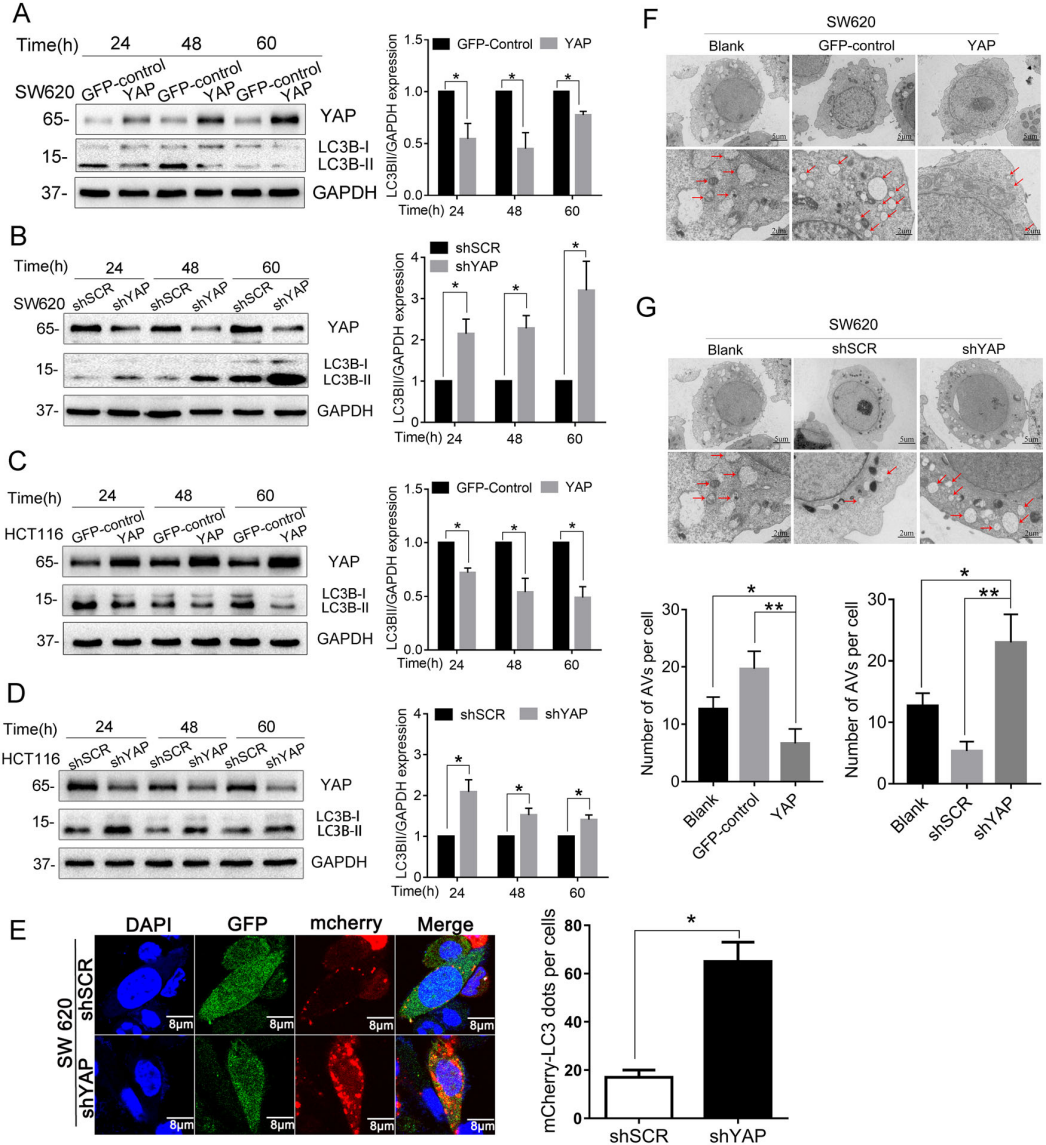
YAP inhibits autophagy in human CRC cells. **A**–**D** SW620 and HCT116 cells with stably overexpressing or knocking down of YAP were cultured in normal conditions for 24, 48, and 60 h. Total proteins were then extracted and subjected to western blot to detect the levels of the two forms of autophagy marker LC3B. **E** SW620 cells stably transfected with shRNA against YAP(shYAP) or scrambled sequence(shSCR) were transfected with mCherry-LC3. After 48 h of transfection, the fluorescence of mCherry-LC3 was determined under fluorescence microscope; GFP is an indicator for cells having shRNA expression. **F**, **G** Transmission electron microscopy was performed on SW620 cells with stably overexpressing or knocking down of YAP to observe autophagy. Arrowheads indicate autophagosomes formed in the cells. Data represent mean ± SD. *P < 0.05, **P < 0.01.

To determine the impact of YAP on autophagy in CRC cells, we first examined the protein levels of LC3B, a hallmark of autophagy, after YAP expression was changed in the cells. Because the LC3B precursor (LC3B-I) is diffusely localized in the cytosol in resting-state cells and can be quickly converted to the lipidated, autophagosome-associated form (LC3B-II) after autophagy induction, the conversion of LC3B-I to LC3-II is widely used to evaluate autophagic activity. As shown in Fig. 1A, C, compared to the GFP-control cells, overexpression of YAP in SW620 and HCT116 cells could decrease the ratio of LC3B-II to LC3B-I; in contrast, knocking down of YAP in the cells by shRNA led to increased LC3B-II protein levels (Fig. 1B, D). These results showed that LC3B-II level is inversely correlated with YAP expression, indicating that YAP negatively regulates autophagy in CRC cells.

Another good indicator of autophagy activation is the punctate accumulation of LC3B, which represents the recruitment of LC3B-II to autophagic vacuoles. To further demonstrate the inhibitory effect of YAP on autophagy, we investigated mCherry-LC3B dot formation in SW620 cell lines with or without YAP knocking down. As shown in Fig. 1E, compared with the shSCR negative control cells, large numbers of punctate mCherry-LC3 proteins were observed in SW620-shYAP cells, suggesting that YAP suppression significantly increases autophagosome formation.

For further confirmation, we performed transmission electron microscopy to visually observe autophagosome formation. As demonstrated in Fig. 1F, autophagic vacuoles in SW620 cells with YAP overexpression were significantly decreased in the perinuclear region compared to control cells, whereas knocking down YAP expression could increase autophagic vacuoles formed in the cells (Fig. 1G). Taken together, by the three complementary experiments, we demonstrated that YAP could inhibit autophagy in CRC cells.

### Bcl-2 mediates YAP-induced autophagy inhibition

**Fig. 2:**
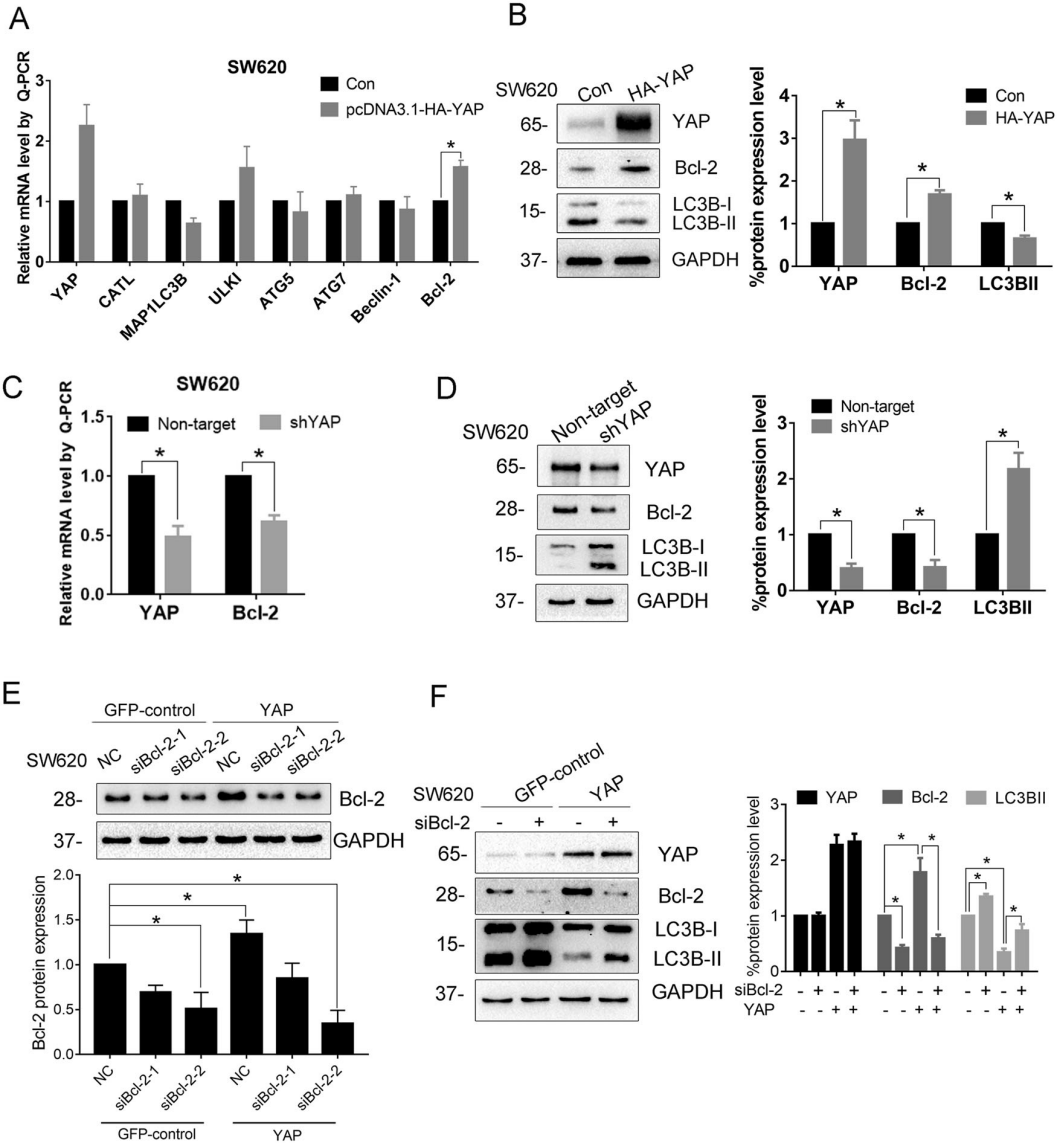
Bcl-2 mediates YAP-induced autophagy inhibition. **A** mRNA levels of autophagy-related genes were analyzed by RT-qPCR in SW620 cells transiently transfected with pcDNA3.1-HA-YAP or control vector for 48 h. **B** Protein levels of Bcl-2 and the two forms of LC3B were investigated after SW620 cells transfected with pcDNA3.1-HA-YAP for 48 h. **C** mRNA and **D** protein levels of *Bcl-2* after knocking down YAP expression in SW620 cells with specific shRNA. Increased ratio of LC3B-II to LC3B-I indicates enhanced autophagy after knockdown of YAP. **E** The YAP-overexpressing (YAP) and control (GFP-control) SW620 cell lines were transfected with 100 nM of Bcl-2-specific siRNA or NC siRNA for 48 h. Western blot was performed to evaluate Bcl-2 silencing efficiency. **F** SW620 cell lines with YAP overexpression (YAP) and control cell line (GFP control) were transfected with 100 nM of Bcl-2-specific siRNA-2 or NC siRNA for 48 h. The protein levels of the two forms of LC3B and Bcl-2 in the cells were investigated by Western blot. Data represent mean ± SD. *P < 0.05.

Next, we proceeded to investigate the mechanism for YAP-inhibiting autophagy. To find out the target genes of YAP, first, we compared the mRNA levels of autophagy-related genes in YAP-overexpressing cells and control cells. The results revealed that *Bcl-2* mRNA level was noticeably upregulated in YAP-overexpressed SW620 cells (Fig. 2A). Since Bcl-2 has been reported to implicate in autophagic process via multiple ways, we further investigated its role in YAP-induced inhibition of autophagy. As shown in Fig. 2B, overexpression of YAP could increase Bcl-2 protein level and suppress the production of LC3B-II. In contrast, knockdown of YAP expression markedly reduced mRNA and protein levels of *Bcl-2* in SW620 cells (Fig. 2C, D); simultaneously, the protein level of LC3B-II was increased. We then asked whether Bcl-2 is a mediator for YAP-induced autophagy inhibition. To illustrate it, we designed two siRNAs that specifically target *Bcl-2* transcripts. The immunoblotting result showed that siBcl-2-2 had a better knockdown efficiency (Fig. 2E). As expected, production of LC3B-II was elevated when Bcl-2 expression was knocked down by siBcl-2-2 in SW620 cells, suggesting enhanced autophagy; more importantly, knockdown of Bcl-2 could attenuate YAP-induced autophagy inhibition (Fig. 2F). Overall, these results strongly suggest that Bcl-2 is a downstream effector for YAP to inhibit autophagy.

### YAP upregulates Bcl-2 expression at the transcriptional level

**Fig. 3:**
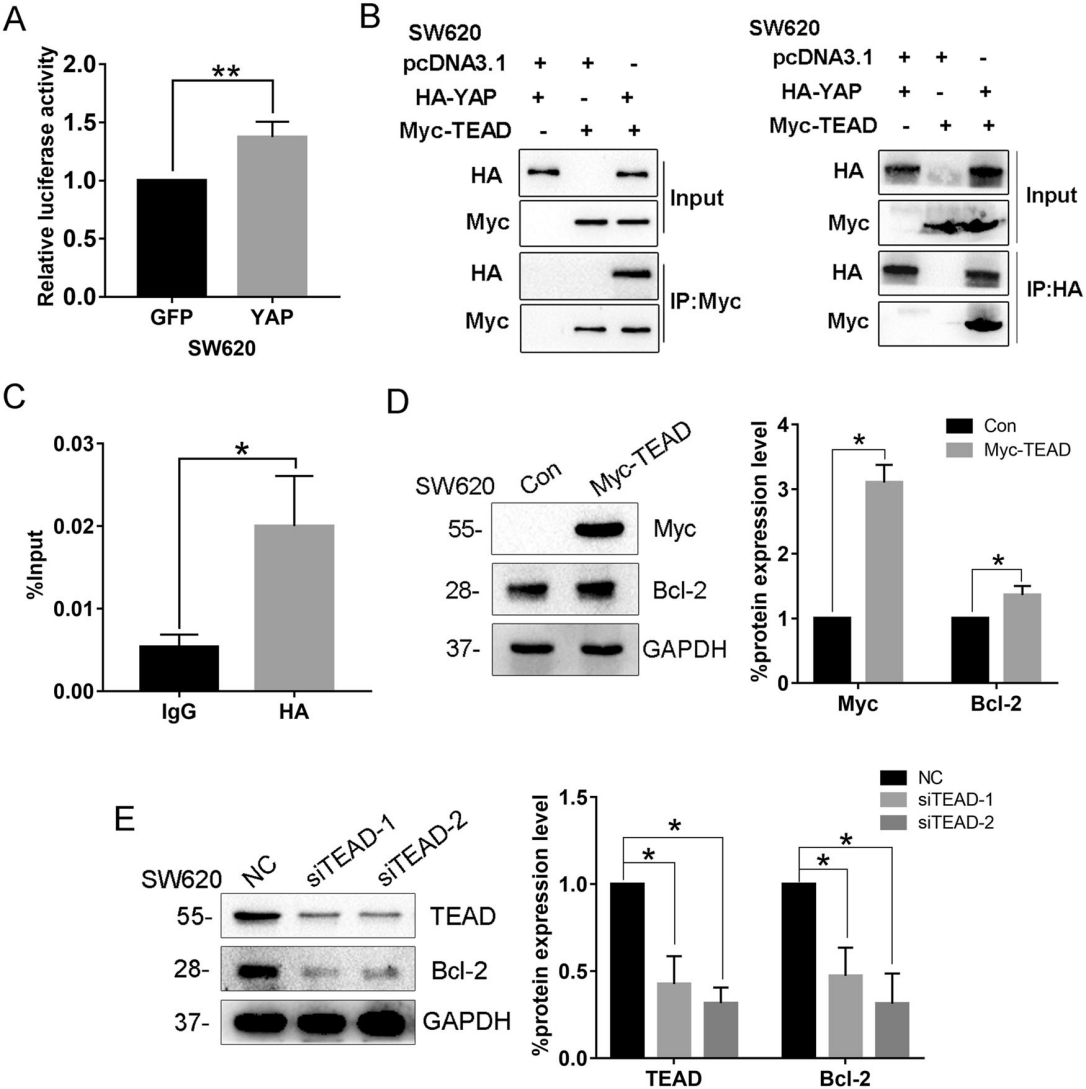
YAP upregulates Bcl-2 expression at the transcriptional level. **A** *Bcl-2* promoter activities in SW620-GFP or SW620-YAP cells were measured using luciferase assay, after the cells were transfected with *Bcl-2* promoter–luciferase reporter constructs. **B** Cell lysates of SW620 cells cotransfected with HA-YAP and Myc-TEAD plasmids were prepared and subjected to Co-IP for investigating the interaction between YAP and TEAD. **C** ChIP-qPCR was performed in SW620 cells transfected with pcDNA3.1-HA-YAP to identify the enrichment of HA-YAP onto *Bcl-2* promoter region. IgG served as an antibody control. **D** Protein levels of Bcl-2 were investigated after SW620 cells transfected with Myc-TEAD for 48 h. **E** SW620 cells were transfected with 100 nM of TEAD-specific siRNA or NC siRNA for 48 h, and then the protein levels of Bcl-2 in the cells were investigated by western blot. Data represent mean ± SD. *P < 0.05, **P < 0.01.

It has already been demonstrated that YAP is a transcriptional coactivator that interacts with other transcription factors, particularly TEAD, to regulate transcription of target genes by binding to distant enhancers and contacting their regulated promoters via DNA looping^18,19^. To address whether YAP could directly regulate Bcl-2 expression, we first performed a luciferase reporter assay in SW620 cells. Using the Bcl-2 promoter–luciferase reporter constructs, we found that Bcl-2 promoter activity is significantly increased in SW620 cells overexpressing YAP compared to the cells expressing GFP (Fig. 3A). Bioinformational analysis indicated that *Bcl-2* gene promoter contains TEAD-binding sites, leading us to speculate that YAP might be recruited to *Bcl-2* promoter by binding with TEAD. Consistent with our speculation, Myc-TEAD could pull down HA-YAP in the Co-IP experiment, and HA-YAP could similarly pull down Myc-TEAD as well (Fig. 3B), suggesting that YAP interacts with TEAD forming a complex in the cells. For further confirmation, we performed ChIP-qPCR to detect the enrichment of YAP on *Bcl-2* promoter. The result showed that YAP was indeed enriched at the promoter region of *Bcl-2* (Fig. 3C). In addition, when TEAD was overexpressed in SW620 cells, Bcl-2 expression could be increased consequently (Fig. 3D), while knockdown of TEAD could decrease the expression of Bcl-2 (Fig. 3E). Taken together, these results indicate that YAP upregulates *Bcl-2* transcription by interacting with TEAD and binding onto *Bcl-2* promoter region.

### YAP inhibits autophagy and promotes growth of CRC cells in vivo

**Fig. 4:**
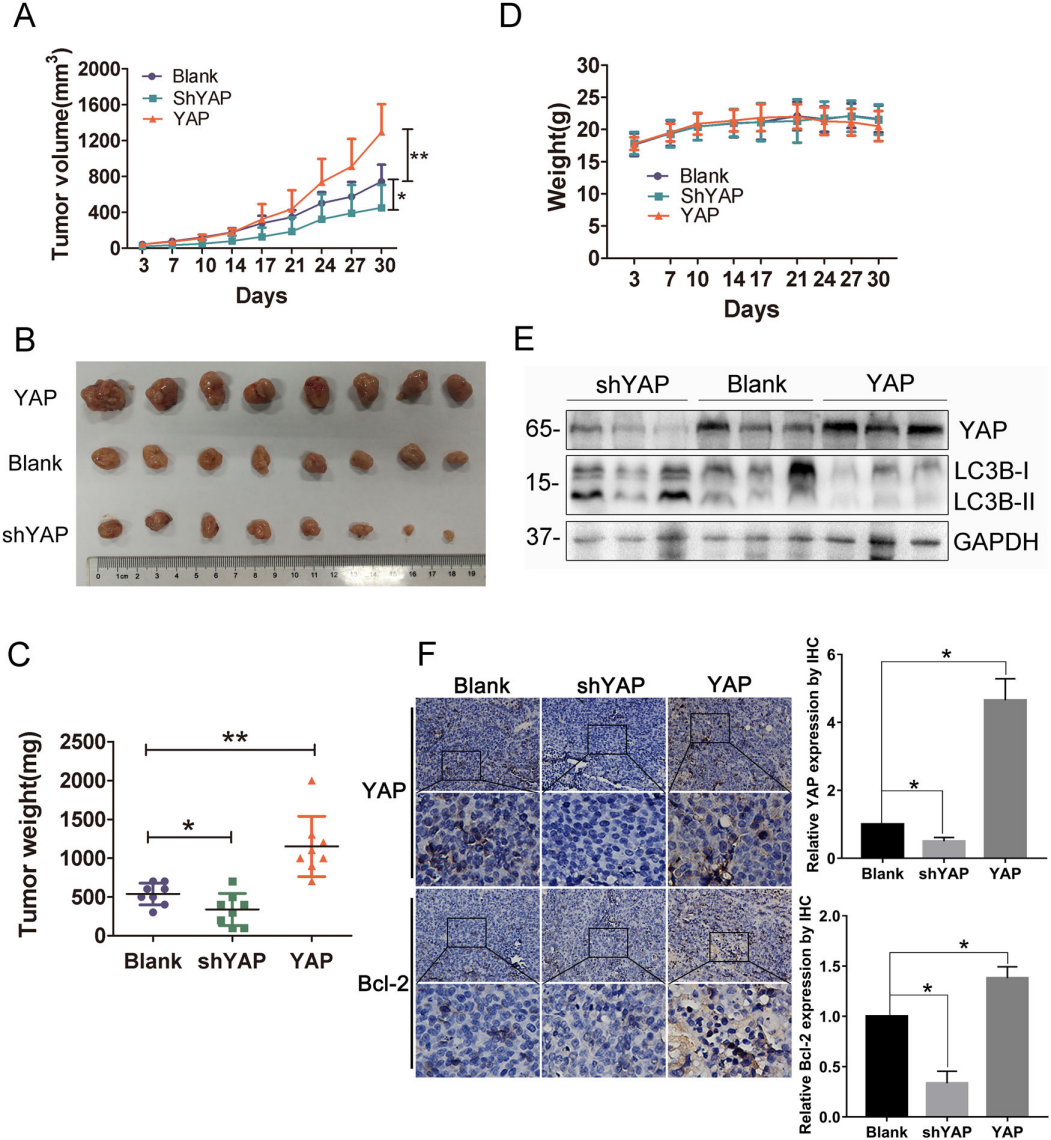
YAP inhibits autophagy and promotes the growth of CRC cells in vivo. SW620 cells with stable YAP overexpression (YAP), knocking down of YAP (shYAP), or control cells (Blank) were subcutaneously injected into nude mice. **A** Tumor size and **D** body weight of the mice were measured twice a week. **B** Tumor tissues were collected from the sacrificed mice nearly 5 weeks after injection of the cells, and **C** weights of the formed tumors were measured for statistic analysis. **E** The expressions of YAP and LC3B in the tumor tissues were measured by western blot, using GAPDH as a loading control. **F** The expression of YAP and Bcl-2 in the tumor tissues was detected by IHC. Data represent mean ± SD. *P < 0.05, **P < 0.01.

Finally, we investigated the effects of YAP on autophagy and tumor growth of CRC cells in vivo. As shown in Fig. 4A, compared to parental blank cells, overexpression of YAP could facilitate in vivo growth of SW620 cells, while knocking down of YAP could inhibit tumor growth in vivo, which is consistent with the effects of YAP on in vitro proliferation of CRC cells (Supplementary Fig. S1). Consistently, tumors harvested from SW620 cells stably overexpressing YAP were the biggest and heaviest, while those formed from SW620 cells stably knocking down YAP expression were the smallest and lightest (Fig. 4B, C). Of note, body weights of the mice bearing different SW620 cells had no significant difference (Fig. 4D). After measuring the levels of LC3B and Bcl-2 in tumor specimens from different groups, we found that tumor tissues formed from YAP-overexpressing SW620 cells have relatively lower LC3B-II levels and higher levels of Bcl-2, whereas those formed from cells knocking down YAP expression have increased levels of LC3B-II and decreased levels of Bcl-2, compared with parental blank cells (Fig. 4E, F). Collectively, these results suggest that YAP could inhibit autophagy and promote the growth of CRC cells in vivo, which is consistent with the findings we found in vitro.

### Activating autophagy can inhibit the YAP-induced CRC progression

**Fig. 5:**
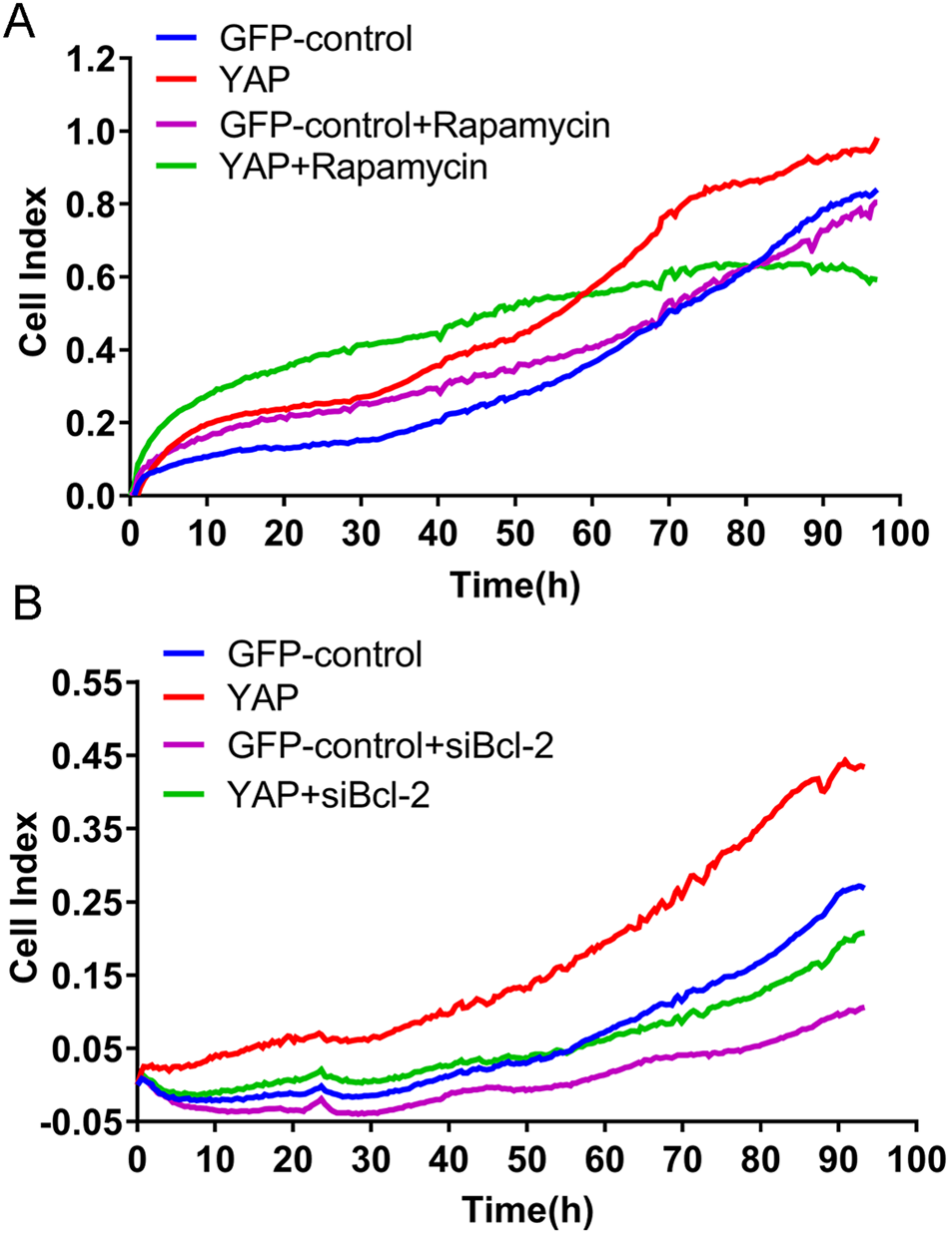
Activating autophagy can inhibit the YAP-induced CRC progression. **A** The YAP-overexpressing (YAP) and control (GFP-control) SW620 cell lines were treated with rapamycin (50 µg/mL) for 90 h, and cell growth was then monitored by RTCA. **B** The YAP-overexpressing (YAP) and control (GFP-control) SW620 cell lines were transfected with 100 nM of Bcl-2-specific siRNA or NC siRNA for 90 h; cell growth was then monitored by RTCA.

To verify the connection between autophagy inhibition and YAP-induced CRC progression, we monitored the in vitro growth of SW620 cells with or without overexpression of YAP by RTCA, after activation of autophagy by 50 µg/mL rapamycin. As expected, the result showed that rapamycin-activated autophagy could alleviate YAP-promoted cell proliferation (Fig. 5A). In addition, we therefore knocked down the expression of Bcl-2 in YAP-overexpressing SW620 cells and observed the effect on cell proliferation. The result also displayed that knockdown of Bcl-2 could inhibit pro-proliferation effect by YAP overexpression (Fig. 5B). Collectively, these results indicate that decreased autophagy contributes to the YAP-induced CRC progression.

## Discussion

Previous studies have reported that increased expression of YAP contributes to cancer progression, including CRC. As a transcription coactivator, YAP forms complexes with other transcription factors to regulate the expression of target genes that are crucial for tumorigenesis. Many mechanisms for YAP tumor-promoting ability have been revealed. For instance, Kang et al. supported that YAP1 exhibits oncogenic property in gastric cancer^9^; Marti et al. revealed that YAP could regulate proliferation, apoptosis, and angiogenesis in human cholangiocarcinoma by functionally interacting with TEAD transcription factors (TEADs)^6^; Zhou et al. found that YAP upregulation could protect BEL cells from chemotherapy in hepatocellular carcinoma via the RAC1–ROS–mTOR pathway^20^. Nevertheless, how YAP functions during CRC progression is not well characterized. Here, by detecting the production of LC3B-II and formation of autophagosomes, we demonstrated that YAP could inhibit autophagy in CRC cells. Autophagy is an evolutionarily conserved process that recycles nonessential cytosolic materials to maintain cellular homeostasis and overcome metabolic stress and nutrient deprivation. It has demonstrated that autophagy contributes to therapeutic resistance; however, excessive or prolonged autophagy causes autophagy-related cell death, leading to tumor regression. The roles of autophagy in CRC are divergent and the underlying mechanisms have not been fully explained yet. Yu et al. reported that autophagy-related pathways are enriched and activated in CRC patients and can induce cancer chemoresistance^21^; Sakanashi et al. suggested that autophagy is upregulated in aggressive CRCs, and p53 mutation may lead to the upregulation of autophagy^22^. Whereas, Wei et al. showed that FAT can inhibit cell invasion, migration, and proliferation by promoting autophagy in colorectal cancer cells^23^. According to our results in this study, basal autophagy seems not to be a helpful event for CRC cell survival, since YAP induced basal autophagy suppression accompanied with increased cell proliferation (Fig. 5 and Supplementary Fig. S1) and in vivo tumor growth (Fig. 4).

More importantly, we revealed that Bcl-2 is the mediator for YAP-induced autophagy inhibition. Bcl-2 is a crucial regulator of autophagy-related cell death by preventing cells from undergoing apoptosis. Overexpression of Bcl-2 contributes to the development of resistance to chemotherapy, radiation, and hormone therapy. In human leukemic cells, downregulation of Bcl-2 triggered autophagy-related cell death^24^; silencing of *Bcl-2* also induced autophagy-related cell death in Bcl-2-overexpressing breast cancer cells^25^. Buchholtz et al. found that breast cancer patients with high Bcl-2 expression had a poor response to chemotherapy compared with those who had less Bcl-2 expression^26^. In this study, we found that Bcl-2 expression is positively correlated with YAP expression; silencing *Bcl-2* expression could alleviate YAP-induced autophagy inhibition with no effect on YAP expression. Mechanistically, YAP upregulates *Bcl-2* expression by interacting with TEAD and binding onto Bcl-2 promoter to initiate transcription process. As a result, elevated Bcl-2 might inhibit induction of autophagy by blocking BECN1^27^.

Taken together, our findings provide evidence that YAP can inhibit autophagy in human CRC cells by transcriptionally upregulating *Bcl-2*, and consequently promote CRC progression. Future studies are needed to discern whether autophagy-related cell death is inhibited by this YAP/Bcl-2 regulatory signaling in CRC cells. Meanwhile, further drug exploration targeting YAP/Bcl-2 would be a potentially effective option against human CRC.

## Materials and methods

### Cell lines and culture

SW620, HCT116, and 293T cells were procured from American Type Culture Collection (ATCC, Manassas, VA, USA). SW620 cells were cultured in Leibovitz’s-15 (L-15) medium (Gibco, Thermo Fisher Scientific, Inc., Waltham, MA, USA) supplemented with 10% fetal bovine serum (FBS, Gibco; Thermo Fisher Scientific, Inc., Waltham, MA, USA) and antibiotics. HCT116 cells were cultured in McCoy’s 5 A medium (Sigma-Aldrich, St. Louis, MO, USA) with 10% fetal bovine serum and antibiotics. 293T cells were maintained in Dulbecco modified Eagle medium (DMEM, Gibco; Thermo Fisher Scientific, Inc., Waltham, MA, USA) supplemented with 10% FBS and antibiotics. All the cells were incubated in a humidified atmosphere with 5% CO_2_ at 37 °C. Possible mycoplasma contamination was routinely measured every 6 months.

### Transfection and stable cell line generation

pcDNA3.1-HA-YAP plasmid, lentivirus vectors pL-GFP-ip, and pLKD-CMV-G&PR-U6-shRNA plasmids were presented by Youbio Co (Changsha, China). YAP coding sequence was cloned into pL-GFP-ip, and the shRNA expression sequence against YAP was cloned into pLKD-CMV-G&PR-U6. Lipo 6000^TM^ transfection reagent (catalog no. C0526, Beyotime, Shanghai, China) was used for transient transfection according to the manufacturer’s instructions. Lentivirus for overexpressing YAP or knocking down YAP expression was packaged in 293T cells. SW620 and HCT116 cells were infected with the lentivirus and selected with puromycin (Santa Cruz) at 48 h post transfection for 4 weeks to obtain corresponding cell lines with stably altered YAP expression.

siRNAs and negative control siRNA were purchased from Shanghai GenePharma Co., Ltd., (Shanghai, China). The sequences of the siRNAs were as follows: siBcl-2-1, 5′-GGAUGACUGAGUACCUGAAdTdT-3′; siBcl-2-2, 5′-GUGAUGAAGUACAUCCAUUdTdT-3′; si-TEAD-1, 5′-CCACGAAGGUCUGCUCUUUTT-3′; si-TEAD-2, 5′-CGCUCUGUGAGUACAUGAUTT-3′; negative control (NC), 5′-UUCUCCGAACGUGUCACGUTT-3′. For knocking down Bcl-2 or TEAD expression, CRC cells were separately transfected with 100 nM of each siRNA using Lipo 6000^TM^ transfection reagent.

### Real-time quantitative PCR (QPCR)

Total RNA was extracted from cells using Trizol reagent (Invitrogen, Thermo Fisher Scientific, Inc., Waltham, MA, USA) according to the manufacturer’s protocol. cDNA was synthesized from 2 µg of total RNA using M-MLV qPCR RT Kit (Toyobo Co., Ltd. Life Science Department, Osaka, Japan). QPCR was performed in an ABI StepOnePlus^TM^ Real-Time PCR System (ABI, Thermo Fisher Scientific, Inc., Waltham, MA, USA) using SYBR^®^ Green Realtime PCR Master Mix (Toyobo Co., Ltd. Life Science Department, Osaka, Japan). Briefly, 100 ng of cDNA template was mixed with 200 nM forward and reverse primers and 10 µl 2×SYBR Green PCR Master Mix. The mixture was adjusted up to 20 μl in volume using ddH_2_O and reacted under the following condition: 95 °C for 3 min, then 40 cycles at 95 °C for 10 s, 60 °C for 30 s, and 72 °C for 20 s. The primers used for QPCR are listed in Table 1. Relative expression levels of the individual genes were calculated using the 2^-ΔΔCt^ method. β-actin was used for normalization. Each experiment was repeated three times.

**Table 1:** Primers used for RT-qPCR assay.

Gene	Primer sequence
*YAP*	F: AGTGGACTAAGCATGAGCAG R: TGTTCATTCCATCTCCTTCC
*CATL*	F: GCATAATCCATTAGGCCACCATT R: CAGATCTGTGGATTGGAGAGA
*MAP1LC3B*	F: TTATTCGAGAGCAGCATCC R: AGGCCTGATTAGCATTGAGC
*ULK1*	F: GGACACCATCAGGCTCTTCC R: GAAGCCGAAGTCAGCGATCT
*ATG5*	F: TTTGAATATGAAGGCACACC R: TGCAATCCCATCCAGAGTTG
*ATG7*	F: GTGCACTGTGAGTCGTCCAG R: GATCTGGTGAGGCACAAGCC
*Beclin-1*	F: AACCTCAGCCGAAGACTGAA R: GACGTTGAGCTGAGTGTCCA
*Bcl-2*	F: GGTCATGTGTGTGGAGAGCG R: AAGCCAGCCTCCGTTATCCT
*β-actin*	F: AGAAAATCTGGCACCACACC R: GGGGTGTTGAAGGTCTCAAA

*F* forward, *R* reverse.

### Western blot

Cell pellets were lysed in CytoBuster Protein Extraction Reagent (Invitrogen) to extract total protein. BCA assay was used to determine the concentration of protein. About 25 µg of total protein were subjected to 8–12% sodium dodecyl sulfate-polyacrylamide gel electrophoresis, and transferred to a PVDF membrane (Millipore, Kenilworth, NJ, USA), followed by blocking with 5% skimmed milk in TBST at room temperature for 1 h. The membrane was then incubated with primary antibodies overnight at 4 °C. The primary antibodies used were anti-YAP (1:1000 dilution, ABclonal), anti-LC3B (1:1000 dilution, Sigma), anti-Bcl-2 (1:1000 dilution, catalog no. AB112, Beyotime, Shanghai, China), and anti-GAPDH (1:1000 dilution, catalog no. AF0006, Beyotime, Shanghai, China). On the second day, after washing the membrane with TBST for three times with 5 min each time, horseradish peroxidase (HRP)-conjugated secondary antibody (1:10,000 dilution, catalog no. E030120-01 and E030110-01; EarthOx, LLC, San Francisco, CA, USA) was used to incubate with the membrane at room temperature for 1.5 h. Detection was performed using a BeyoECL Plus kit (catalog no. P0018S, Beyotime, Shanghai, China). The defined sections of the film were scanned for image capture, and quantified using Adobe Photoshop software (CS4, Adobe Systems Incorporated, USA) and ImageLab software (Bio-Rad, USA).

### Luciferase reporter assay

The luciferase vectors were obtained from Addgene Company (USA). SW620 cells with stable overexpression of YAP or control cells were separately seeded in a 24-well plate. On the second day, the cells were transfected with luciferase reporter construct containing the promoter region of *Bcl-2*. After 48 h of transfection, firefly and Renilla luciferase activities were measured using the dual-luciferase kit (Promega, WI, USA) according to the manufacturer’s protocol.

### Transmission electron microscope

Cells were collected by centrifugation after 48 h of culture under normal conditions, then fixed with 2.5% glutaraldehyde in 0.1 M cacodylate buffer (pH 7.4) for 2 h. The prepared cells were delivered to ServiceBio company (Wuhan, China) for transmission electron microscopic analysis.

### Immunofluorescence

Cells were transfected with mCherry-LC3B plasmid for 48 h, and then fixed in 4% paraformaldehyde for 20 min. After staining of DAPI for 5 min, the cells were observed and photographed under a fluorescence microscope.

### ChIP-qPCR assay

A total of 4 × 10^6^ SW620 cells were harvested and cross-linked by formaldehyde at a final concentration of 1%. After stopping cross-link with glycine, chromatin was sheared to 100–300 bp with sonication. The anti-HA-Tag antibody (Abcam, ab9110) and protein G beads were applied to pull down the target protein. Then the protein was digested with proteinase K. DNA immunoprecipitated by the target protein was harvested and purified, and detected by qPCR. The sequences of the primers are forward: 5′-AAAATCATAATTTGGTGTGCTTTTCTGG-3′, reverse: 5′-GAACTCCTGACCTCGTTATCCG-3′. Input DNA was used for normalization. The assay was completed in Wuhan GeneCreate Biological Engineering Company.

### Co-immunoprecipitation (Co-IP)

SW620 cells were transfected with the HA-YAP and/or Myc-TEAD plasmids using Lipo 6000^TM^ transfection reagent. Cells were washed twice with PBS and lysed on ice for 30 min using lysis buffer. The lysates were collected by centrifugation at 13,000 rpm for 10 min at 4 °C and precleared by incubation with protein G Sepharose beads (Abmart) for 3 h at 4 °C under continuous rotation. Following centrifugation, primary antibodies were added to the supernatants and incubated for 4 h at 4 °C under continuous rotation, and then, protein G Sepharose beads were added and incubated overnight at 4 °C under continuous rotation. Finally, the beads were collected and washed three times with washing buffer (5% sucrose, 5 mM Tris-HCl [pH 7.5], 5 mM EDTA, 500 mM NaCl, and 1% Triton X-100), followed by denaturation at 100 °C for 5 min in 2 × SDS protein loading buffer and detection by western blot.

### Real-time cellular analysis (RTCA)

Cells treated with 50 µg/mL rapamycin (Sigma) or transfected with siBcl-2 were separately seeded in a 16-well E-plate and incubated at 37 °C. Cell proliferation was continuously monitored by Agilent xCELLigence RTCA eSight for 90 h according to the manufacturer’s protocol.

### Immunohistochemistry

The tumor tissues were deparaffinized in xylene. The primary antibodies against YAP (ABclonal) or Bcl-2 (Beyotime, Shanghai, China) were used at suitable concentrations and incubated overnight at 4 °C. The intensity of staining was calculated according to the immunoreactive score system (IRS).

### Human CRC xenograft experiments

Five-week-old nude immunodeficient mice (nu/nu) (weighing ~16 g) were purchased from Hunan SJA Laboratory Animal Co., Ltd. (Changsha, China), and maintained and monitored in a specific pathogen-free environment (temperature, 22–24 °C, barrier environment, 12-h dark/12-h light cycle, sterile water, and full nutritive feed). All animal studies were conducted according to protocols approved by the Hubei University of Medicine Animal Care and Use Committee, complying with the rules of Regulations for the Administration of Affairs Concerning Experimental Animals (approved by the State Council of China). The mice were divided randomly into three groups (5 females and 5 males in each group), which were injected with SW620-shYAP, SW620-blank, and SW620-YAP stable cell lines, respectively. In total, 1 × 10^7^ of 100-µl L-15 medium suspended cells were injected subcutaneously into the right flank of each mouse. Caliper measurements of the longest perpendicular tumor diameters were performed twice a week to calculate the tumor volume using the following formula: (4π/3) × (width/2)^2^ × (length/2). The mice were monitored for about 5 weeks. Animals were sacrificed when tumors reached 1.5 cm in diameter, or paralysis or major compromise in their quality of life occurred. At the time of death, tumors were excised for further examination. No blinding was done in the animal experiment.

### Statistical analysis

Continuous data from three independent experiments were presented as mean ± SD, unless noted otherwise. Statistical differences were determined using the Student’s *t* test of unpaired data when comparing two groups or one-way analysis of variance and Bonferroni posttest when comparing multiple groups. Statistical significance was defined as *P* < 0.05.

## Supplementary information

Supplementary Figure 1

Supplementary Figure 1 Legend

## Data Availability

The datasets used and/or analyzed in the current study are available from the corresponding author on reasonable request.
