# Supplementary figures and images for: YAP inhibits autophagy and promotes progression of colorectal cancer via upregulating Bcl-2 expression
Source: Cell Death Dis. 2021 May 7;12(5):457. doi: 10.1038/s41419-021-03722-8 (PMC8105309; doi:10.1038/s41419-021-03722-8)

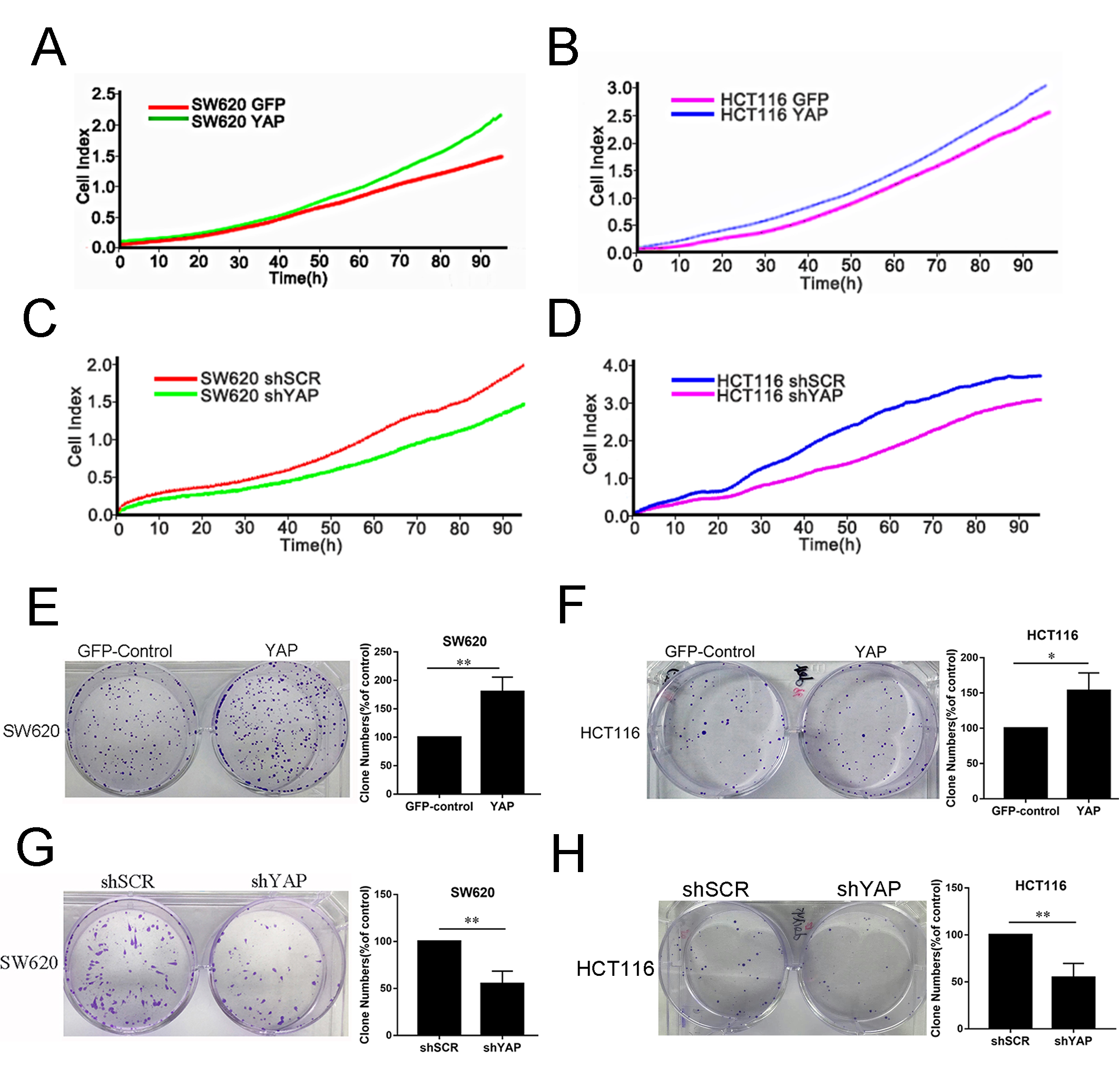

Supplement: Supplementary file 1 — Supplementary Figure 1 [file 41419_2021_3722_MOESM1_ESM.tif]
