# Supplementary material for: YAP inhibits autophagy and promotes progression of colorectal cancer via upregulating Bcl-2 expression
Source: Cell Death Dis. 2021 May 7;12(5):457. doi: 10.1038/s41419-021-03722-8 (PMC8105309; doi:10.1038/s41419-021-03722-8)
Supplement: Supplementary file 2 — Supplementary Figure 1 Legend [file 41419_2021_3722_MOESM2_ESM.docx]

**Figure S1. YAP promotes in vitro proliferation of CRC cell.**

**(A-D)** SW620 and HCT116 cells with stably overexpressing or knocking down of YAP were separately seeded into 16-well E-plate. Cells were cultured in normal conditions and cell index was continuously monitored by Agilent xCELLigence RTCA eSight for 90 h to observe cell proliferation. **(E-H)** The colony formation assays of SW620 and HCT116 cells with stably overexpressing or knocking down of YAP. Cells were suspended in 2 ml of L-15 or McCoy’s 5A and seeded into 6-well plates. After 2 weeks, cell colonies were stained with 0.2% gentian violet and counted under light microscope. Data represent mean±SD. **P*<0.05, ** *P*<0.01.
